# The Role of Plant-Derived Bioactive Compounds in Mitigating Oxidative Stress

**DOI:** 10.3390/foods15010108

**Published:** 2025-12-30

**Authors:** Aslıhan Tüğen, Claudia Lavinia Buruleanu

**Affiliations:** 1 Department of Food Engineering, Institute of Sciences, Afyon Kocatepe University, 03200 Afyonkarahisar, Türkiye; 2 Department of Food Engineering, Faculty of Environmental Engineering and Food Science, Valahia University of Targoviste, 13 Sinaia Alley, 130004 Targoviste, Romania

**Keywords:** oxidative stress, plant-derived bioactive compounds, polyphenols, flavonoids, terpenes

## Abstract

Oxidative stress arises from an imbalance between reactive oxygen species (ROS) and antioxidant defense mechanisms and disrupts the structural integrity of macromolecules such as lipids, proteins, and DNA. This biochemical imbalance triggers the pathogenesis of cardiovascular and neurodegenerative diseases and leads to lipid oxidation and quality degradation in food systems. Plant-derived bioactive compounds (BACs) such as polyphenols and terpenes develop versatile molecular strategies to mitigate this oxidative damage. In addition to their direct radical scavenging effects, polyphenols stimulate the synthesis of endogenous antioxidant enzymes such as superoxide dismutase (SOD) and catalase (CAT) by activating the Nrf2–Keap1 signaling pathway. Terpenes, on the other hand, create a specialized protective shield in lipid-based matrices through “chain-breaking” reactions and a “slingshot” mechanism that externally halts the oxidation of γ-terpinene. In food engineering applications, these compounds meet the demand for “clean-label” products by providing alternatives to synthetic antioxidants such as BHA and BHT. Specific terpenes, such as carnosic acid, demonstrate higher performance in inhibiting lipid oxidation compared to their synthetic counterparts. Although BAC use extends the shelf life of products while maintaining color and flavor stability, potential interactions with protein digestibility necessitate dosage management. From a clinical perspective, these compounds suppress inflammatory responses by inhibiting the NF-κB pathway and contribute to the prevention of chronic diseases by modulating the gut microbiota. This review evaluates the capacity of BACs to manage oxidative stress in food preservation technologies and human health through a mechanistic and application-based approach.

## 1. Introduction

Oxidative stress results from the disruption of the balance between reactive oxygen species (ROS) and antioxidant defense mechanisms, which damages the structural integrity of lipids, proteins, and DNA. Such cellular damage has played an important role in the development of chronic diseases such as cardiovascular and neurodegenerative diseases, cancer, and diabetes.

In redox metabolic processes, reactive free radical species such as singlet oxygen (^1^O_2_), superoxide anion (O_2_^−^·), hydrogen peroxide (H_2_O_2_), or hydroxyl radical (OH·) are generated. Biomolecules from cells react strongly with these ROS, transforming into free radicals that trigger a chain reaction. As a result, the biomolecules involved in the organization and functioning of the cell are degraded. The acute and long-term action of free radicals in living tissues leads to the disruption of many metabolic pathways and modifies the properties of cellular structures with pathological consequences.

Cell membranes, which contain easily oxidizable polyunsaturated fatty acids, are the most exposed to the action of free radicals. The structural modifications of these fatty acids have the effect of changing the characteristics of the membranes, mainly their fluidity and semipermeability. Some possible consequences of tissue lipid peroxidation include changes in low-density lipoproteins (LDLs), increased risk of atheroma formation, altered platelet function, an altered arachidonic acid cascade, protein polymerization, and DNA mutations. The free radical and peroxide neutralization reactions, in which antioxidants play an important role, represent a general protective mechanism against increased lipoperoxidation, which is considered to have repercussions on health by favoring numerous pathologies.

The human body has a wide range of means to control peroxidation and rapidly inhibit, eliminate, and/or inactivate the free radical generators (Table 1). Scavenging enzymes, namely, primary and secondary antioxidants, are able to provide protection against free radical-induced degradation.

The great effectiveness of antioxidants lies in their synergism, each functioning according to different mechanisms and at various levels of the chain of evolution of the free radicals in the body. Coming from food, the non-enzymatic antioxidants comprise a large group of compounds, including plant-derived bioactive compounds.

Bioactive compounds of plant origin, especially phenolic compounds (polyphenols including flavonoids) and carotenoids (terpenoid pigments), can neutralize the harmful effects of ROS and reduce the level of oxidative damage. Thus, these compounds have been reported to have a protective function in reducing the likelihood of oxidative stress-related diseases [1].

Oxidative processes pose a serious problem not only for human health but also for food quality. Lipid oxidation in foods leads to spoilage of products, loss of flavor and aroma, reduction in nutritional value, and the emergence of toxic compounds. Therefore, in the field of food engineering, it is of great importance to keep oxidation under control in order to preserve the flavor, nutritional content, and safety of products. Today, increasing consumer demand for “clean-labeled” foods strengthens the interest in the substitution of synthetic antioxidants such as BHA and BHT with natural alternatives. Plant extracts rich in polyphenols, flavonoids, and terpenoids show high antioxidant capacity in various food systems. These compounds prevent oxidative degradation of lipids and other macromolecules by neutralizing free radicals [2].

In the current review, we discuss oxidative stress, types of antioxidants, and some representatives of plant-derived bioactive compounds (BACs), namely polyphenols, flavonoids, and terpenes. For healthcare professionals, it is important to understand their mechanisms of action. For the food industry, it is important to identify sources of phytochemicals that could replace the synthetic antioxidants in order to develop new food products and understand how BACs could be effectively extracted and integrated in the food matrix. Finally, for consumers, it is important to distinguish between the scientific evidence and the commercial hype when they are choosing food based on their antioxidant content.

## 2. Materials and Methods

A review of the relevant literature was conducted to achieve the objectives of this study. The Boolean search strategy was used for searching the literature in the prominent databases Web of Science (WoS), Scopus, and Google Scholar. We focused on specific keywords from different groups: “plant-derived bioactive compounds”, “polyphenols”, “flavonoids”, and “terpenes” in combination with other keywords such as “antioxidants”, “mechanism of action”, “encapsulation”, and ‘’extraction’’. The Boolean operators “AND” and “OR” were employed. The interval of publication and type of publication were used as filters. All the references included in this paper were selected and reviewed in agreement with the proposed objectives.

## 3. The Issue of Oxidative Stress

Oxidative stress is defined as a condition that arises from the disruption of the balance between the production of reactive oxygen species (ROS) and reactive nitrogen species (RNS) within the cell and the antioxidant defense systems responsible for eliminating them, ultimately leading to cellular damage [3]. The disturbance of the equilibrium between the production of ROS and endogenous antioxidant defense systems constitutes the primary triggering mechanism that results in the loss of redox homeostasis in cells and initiates oxidative stress [4,5]. When the rate of ROS production increases due to factors such as inflammation, hyperglycemia, hypoxia, toxic agents, or mitochondrial dysfunction or when the expression and activity of antioxidant systems decrease owing to genetic, environmental, or age-related causes, this balance is disrupted, and the antioxidant capacity is exceeded [6,7]. As a consequence of this disequilibrium, cellular redox signaling is impaired, ROS levels rise beyond the physiological signaling range, and the cell becomes subjected to a pathological oxidant burden defined as oxidative stress [4,8].

When oxidative stress develops, ROS and RNS induce cumulative damage to nucleic acids, proteins, and lipids, thereby disrupting cellular integrity in multifaceted ways [9,10]. At the DNA level, oxidative modification of certain bases, single- and double-strand breaks, and replication errors emerge, increasing mutational load and predisposing to tumor development and degenerative processes [11,12]. Thiol groups in proteins undergo oxidation, carbonylation increases, and structural and functional impairments occur, leading to reduced catalytic efficiency of enzymes and altered intracellular signaling pathways [3,9]. Polyunsaturated fatty acids in cell membranes undergo lipid peroxidation, membrane fluidity and permeability are disturbed, ion balance is altered, and consequently calcium homeostasis becomes compromised, triggering cell death pathways toward necrosis or apoptosis [12,13].

Oxidative stress also plays a role in the pathophysiology of chronic diseases. In cardiovascular diseases, this process disrupts the proper functioning of vascular walls (endothelial dysfunction) [14,15], accelerates the formation of atherosclerotic plaques (arterial hardening) through the oxidation of low-density lipoproteins (LDLs) [4], and reduces nitric oxide bioavailability, thereby predisposing to hypertension and heart failure [16,17]. Similarly, in neurodegenerative disorders such as Alzheimer’s and Parkinson’s disease, oxidative stress damages vulnerable neuronal populations [16,18], enhances abnormal protein accumulations (such as amyloid and tau) [19], accelerates neuronal loss [15], and intensifies neuroinflammation (the inflammatory response within the brain) [20,21]. Thus, oxidative stress serves as a key mechanism in the onset and progression of these diseases [15,17,21].

Because oxidative stress has different intensities and manifestations in different cell types, it is crucial to study the biomarkers of oxidative stress. Five types of biomarkers related to oxidative stress have been defined, and it is useful to measure more than one biomarker to understand their clinical relevance [22].

## 4. Antioxidants

Modern lifestyles, including the industrialized lifestyle, expose people to a variety of exogenous harmful factors, such as air pollution, chronic stress, and unhealthy dietary habits. These factors can damage cells and lead to advancement of various maladies [23,24]. ROS generation is higher in ultra-processed food consumers and individuals living in contaminated areas [5,23]. Western diets, high-calorie diets, and low consumption of plant-based fiber cause oxidative stress and are associated with diseases [5].

Antioxidant systems are present in cells in order to protect them against ROS [24]. Antioxidants encompass a wide range of compounds, extremely diverse in biological and chemical terms [25]. Biological antioxidants avoid oxidative stress based on their capability to delay or to prevent the oxidation of a substrate.

Antioxidants or ‘’scavengers’’ exhibit activities to decrease oxidants by donating or accepting electrons to radicals, transforming themselves into less reactive radicals [5]. The scavenging ability of antioxidants against free radicals to prevent cellular damage differs from one compound to another. The antioxidant activity depends by the number of active groups (−OH or NH_2_) and the position of these functional groups.

Antioxidants can be classified based on different criteria. If their nature is discussed, we are talking about natural and synthetic antioxidants. The last ones include BHA, BHT, tert-butylhydroquinone (TBHQ), propyl gallate, and 4-hexylresorcinol. The natural antioxidants are classified as enzymatic and non-enzymatic antioxidants. Superoxide dismutase (SOD), catalase, glutathione reductase, and glutathione peroxidase belong to the group of enzymatic antioxidants. Glutathione and coenzyme Q are known as endogenous non-enzymatic antioxidants. Both enzymatic and non-enzymatic endogenous antioxidants are products of cellular metabolism and act to enhance cellular antioxidant defenses to reduce ROS [5].

Polyphenols, vitamins, and carotenoids are dietary/exogenous non-enzymatic antioxidants. Flavonoids, phenolic acids, stilbenes, and lignans constitute the primary classes of polyphenols [26,27]. The consumption of fruits, vegetables, and their derived products represents the main source of intake of exogenous antioxidants. Along with endogenous antioxidants, dietary antioxidants can maintain redox homeostasis, helping to maintain low ROS concentrations [5,28].

The possible action of exogenous dietary antioxidants and their positive effects on human health are still disputable due to several factors, such as an insufficient knowledge regarding the mechanisms underlying the interaction of the dietary antioxidants in the body. To achieve a precise conclusion regarding the relationship between antioxidant-containing foods and their health benefits, additional investigations and observations are still needed, while numerous challenges must be overcome [25,26,27,29]. The therapeutic effects of antioxidants depend not only on their chemical structures, which are responsible for their mechanisms of action and biochemical pathways, but also on the optimal dose and the local environment factors (i.e., pH, ROS concentration, and presence of other bioactive compounds) [25]. The source of antioxidants has an impact on their efficacy, and other nutrients from the food matrix often act synergistically with these antioxidants.

## 5. Plant-Derived Bioactive Compounds (BACs)

Bioactive molecules are found in high amounts in natural products and exhibit a range of potential applications. Almost all types of living beings produce BACs, including various microorganisms and animals [24].

Polyphenols, vitamins, bioactive peptides, dietary fibers, biogenic amines, and carotenoids are included in the group of plant-derived bioactive compounds. Originating from metabolism, they are generally known as secondary metabolites and recognized for their promising therapeutic properties [30].

In recent years, people have shown considerable interest in phytochemical compounds in foods, which are promoted as antioxidants and healthful agents in the popular literature, often in contrast to harmful oxidants [26]. There have been many research articles and reviews focused on various aspects of antioxidants from different dietary sources, such as antioxidant assays and in vitro and in vivo mechanisms of action.

The antioxidant activity depends on the chemical structure of the plant-based phytochemicals, which are measured in vitro using antioxidants assays sensitive to the metadata associated to the sample. Cupric ion reducing antioxidant capacity (CUPRAC), ferric reducing antioxidant power (FRAP), oxygen radical absorbance capacity (ORAC), DPPH scavenging activity, Folin–Ciocalteu reducing capacity, Trolox equivalent antioxidant capacity (TEAC), glutathione peroxidase (GSHPx) estimation, and superoxide dismutase (SOD)-based methods are examples of assays with advantages but also with some limitations if their specificity and equivalence are discussed. Advanced analytical techniques (i.e., spectroscopy and chromatography) can provide an accurate evaluation of phytochemicals in terms of content and efficacy [31].

The bioaccessibility and bioavailability of antioxidants must be taken into account to evaluate the biological activity of phytochemicals. BACs must be released from the food matrix in an absorbable form into the stomach/intestine, with the matrix interaction influencing the bioaccessibility. The bioavailability of BACs, specifically their absorption into the blood stream, can be affected by biological membranes and the biological environment inside the intestine [26].

### 5.1. Polyphenols as Antioxidants

Polyphenols constitute a large group of secondary plant metabolites recognized for their high antioxidant capacity and include subgroups such as phenolic acids, flavonoids, stilbenes, and tannins. These compounds contribute to the direct control of oxidative stress by neutralizing reactive oxygen species (ROS) such as hydroxyl radicals and superoxide anions before they damage cells [32].

The protective effect of polyphenols on oxidative stress is mediated through multifaceted and interconnected biochemical mechanisms. First, they show strong antioxidant properties thanks to their capacity to directly remove reactive oxygen species and nitrogen species (RNS) [33,34]. Multiple hydroxyl groups in their chemical structure play an important role in electron donation that facilitates the neutralization of free radicals [35]. In addition, polyphenols contribute to the reduction of free radical formation by chelating metal ions [34]. Their effects are not only limited to direct scavenging processes, but they also reduce ROS production by suppressing the activity of oxidative enzymes such as xanthine oxidoreductase [36]. These biochemical interactions help maintain the balance of general oxidoreductase enzyme systems [37].

**Figure 1:**
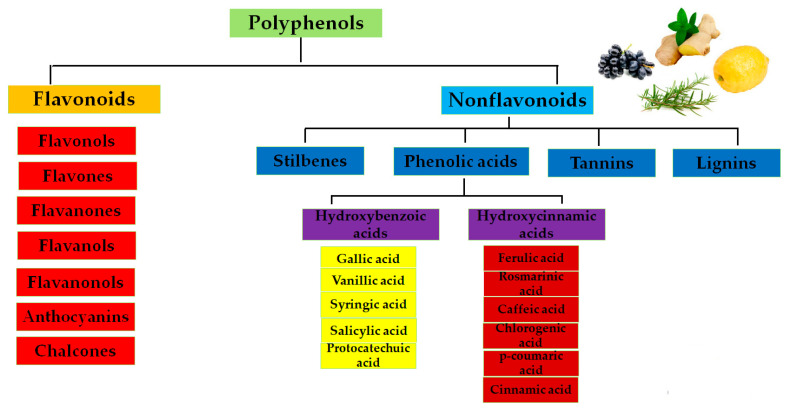
Classification of polyphenols.

Polyphenols can be classified as flavonoids and nonflavonoids [38] as shown in Figure 1.

The processes by which antioxidants neutralize free radicals are explained through multiple reaction pathways. In hydrogen atom transfer (HAT), at certain temperatures, a storage atom is transferred in the radical direction, and the radical reactivity between them is extinguished. This pathway proceeds in a single step, and its kinetic and thermodynamic structure is mostly discussed in terms of the O–H breaking energy (BDE). The proton-coupled electron transfer (PCET) approach is based on the premise that protons and electrons appear in their modern form as a quantum-level coupled structure. In this mechanism, proton and electron motion proceed within a single coordinated framework. In sequential proton loss electron transfer (SPLET), at certain temperatures, an initial tunable, humidifying proton loss occurs, and an intermediate species is formed. This subsequently transfers an electron to the radical, strengthening the radical characteristics. This pathway is more suitable in polar solvents like water, as the solvent stabilizes the anion through solvation. In single electron transfer—proton transfer (SET-PT) pathways, electron distribution occurs before the radical transforms into a cation. Then, a more stable radical form is formed through proton loss. This pathway involves high ionization potential, and researches favoring broad polarity tend to pursue this route less frequently. This reciprocal HAT progresses with a direct product policy. SPLET is becoming more dominant in polar solvents. SET-PT is processed in a more limited number of applications due to its energy units. PCET, which is the simultaneous configuration of proton and electron transport, is given as a model [39].

Polyphenols not only exert direct antioxidant effects, but also strongly activate the natural defense mechanisms of the organism. These compounds act on intracellular signaling pathways by increasing the activity of proteins that regulate gene expression, such as Nrf2 (nuclear factor erythroid 2-related factor 2) [35,36]. Activation of the Nrf2–Keap1 pathway increases the synthesis and function of many antioxidant enzymes such as superoxide dismutase and catalase [33,40]. In particular, stimulation of the Nrf2/GPx4 pathway plays a fundamental role in cellular protection processes [41]. The glutathione peroxidase 4 (GPx4) enzyme targets lipid peroxides formed in cell membranes and ensures the reduction of these compounds. Thus, the structural integrity of the cell membrane is maintained, and the iron-dependent cell death process called ferroptosis is prevented [41].

The protective effects of polyphenols are closely related to anti-inflammatory mechanisms. These plant compounds control cellular communication pathways that initiate inflammation [35]. In particular, they inhibit the activation of proteins (transcription factors) that activate genes such as NF-κB [34]. This inhibition reduces the synthesis and release of cytokines that increase inflammation [34,36]. Polyphenols interact with key proteins involved in stress response such as PI3K–Akt and IL-17 [42]. Polyphenols exhibit anti-inflammatory effects by regulating intracellular signaling and gene expression profile [43].

Polyphenols contribute to the maintenance of mitochondrial health and function at the cellular level. They support energy production by repairing or revitalizing mitochondrial functions and reduce the levels of mitochondria-derived reactive oxygen species in this process [37,44]. Polyphenols stimulate autophagy mechanisms, enabling the clearance and recycling of damaged cellular components [44]. These integrated functions protect fats, proteins and DNA from stress-induced damage [33]. In addition, polyphenols improve nitric oxide homeostasis and contribute to the regulation of eNOS enzyme activity [36,43]. The correlation between the mechanism of action of polyphenols and their effects is shown in Table 2.

### 5.2. Flavonoids and Oxidative Stress

Flavonoids constitute an important subgroup of polyphenols, which are highly abundant in fruits, vegetables, tea, and cocoa. Flavonols such as quercetin and flavanols such as catechins and anthocyanins are included in this group. These compounds exhibit strong antioxidant properties both directly and indirectly. Flavonoids donate electrons or hydrogen atoms to neutralize reactive oxygen species (ROS). Thus, flavonoids ensure the interruption of free radical chain reactions in cells. They also contribute to the reduction of oxidative stress by regulating the antioxidant enzyme systems of the organism [32].

Flavonoids, which are widely found in the plant kingdom and constitute one of the largest classes of phenylpropanoid derivative secondary metabolites, are defined as polyphenolic compounds with a C6-C3-C6 carbon skeleton [45]. This broad chemical family, with approximately 10,000 identified members, is divided into various subclasses such as flavones, flavonols, flavanones, flavanonols, flavanols (catechins), anthocyanins, and chalcones, depending on their structural properties, oxidation levels, and substitution patterns [46,47]. While most flavonoid classes exhibit a 2-phenylchroma structure, chalcones differ structurally from other groups because they lack a central heterocyclic ring and exhibit an open-chain structure [48]. The spectrum of biological activity exhibited by these compounds is directly related to the specific properties in their molecular structure. In particular, the hydroxyl group at the third carbon position, the double bond between the second and third carbons, and the carbonyl group at the fourth position are among the key elements that determine antioxidant capacity and free radical scavenging effects [49]. From the perspective of plant physiology, flavonoids not only attract pollinators by providing pigmentation in flowers, but also activate defense mechanisms that protect plants against various biotic and abiotic stress factors such as UV radiation, drought, salinity, and pathogen attacks [47]. When their effects on human health are examined, it is seen that dietary flavonoids reduce the risk of atherosclerosis and thrombosis by inhibiting low-density lipoprotein (LDL) oxidation and platelet aggregation, thus exhibiting a strong cardioprotective function [50]. It has been reported that phenolic compounds, which are concentrated especially in fruits and nuts of the Rosaceae family, contribute to the prevention of chronic metabolic diseases such as type 2 diabetes by regulating insulin resistance and glucose intolerance [51]. In addition, the literature highlights that flavonoids have anti-inflammatory, antiviral, and neuroprotective properties and exhibit anticancer effects through mechanisms such as cell cycle arrest or apoptosis induction [52,53]. Pharmacological research has shown that chalcone derivatives and some synthetic flavonoids, in particular, exhibit much higher antibacterial activity compared to standard antibiotics and can be effective even against bacterial strains that have developed multidrug resistance [54]. However, the variability of flavonoid concentrations in plant matrices depending on environmental factors and the diversity of their chemical structures make the isolation of these compounds difficult, and the use of optimized methods such as ultrasound-assisted solvent extraction has become a necessity for obtaining high-purity and high-yield substances [45,53].

Flavonoids are effective in keeping oxidative stress under control, mainly through their ability to directly capture free radicals [55,56]. These compounds neutralize reactive oxygen species that can damage cell structure [57]. This neutralization power of flavonoids is based on their unique chemical structure [58]. They show a pronounced binding tendency especially against hydroxyl radicals with high reactivity [59]. This feature is due to structural elements such as catechol group, conjugated double bonds between certain pairs of atoms and hydroxyl groups in their molecular structure [58,59]. In addition, some additional structures, such as a gallate group, further strengthen the free radical scavenging capacity of flavonoids when added to the molecular structure [59].

The protective effect of flavonoids against oxidative stress is not limited to the direct elimination of harmful molecules. These compounds also contribute to the regulation of endogenous and enzyme-based defense systems of the organism [56]. Flavonoids increase the activity of essential antioxidant enzymes such as superoxide dismutase (SOD), catalase (CAT), and glutathione peroxidase (GPx) [57,60]. In studies, it has been determined that certain flavonoid species, such as diosmin and diosmetin, contribute to the normal functioning of SOD and CAT enzymes that lose their function after exposure to hydrogen peroxide (H_2_O_2_), even if they do not show direct radical scavenging function [61]. This effect strengthens the defense system responsible for detoxification of reactive oxygen species [56].

This regulatory effect of flavonoids on the body’s enzymatic defense is mediated by intracellular signal transduction pathways. These compounds initiate the antioxidant response by stimulating the key transcription factor Nrf2, which controls the activation of protective genes [60,62]. Activation of the Nrf2 pathway enables the organism to synthesize more antioxidant enzymes [60]. The dual effect of flavonoids in both direct radical scavenging function and strengthening cellular defense systems helps to maintain the redox balance of cells [57]. As a result of this process, there is a decrease in the level of intracellular reactive oxygen species [63,64] and the amount of malondialdehyde (MDA), an indicator of cell membrane damage [61]. These mechanisms increase the resistance of cells to oxidative damage [55] and contribute to restore the integrity of mitochondrial membranes [64]. The action of flavonoids as antioxidants is outlined in Table 3.

### 5.3. Terpenes and Oxidative Stress

Oxidation of lipophilic components in food reduces the quality of food and shortens its shelf life [65,66]. This process leads to the formation of peroxides in the first stage, followed by the formation of secondary harmful compounds such as malondialdehyde (MDA) [67]. Terpenes, which are considered as a natural alternative to synthetic antioxidants, have an important potential to increase the resistance of foods against oxidative deterioration [67,68]. Terpenes have the capacity to neutralize reactive oxygen species formed in oily environments through different mechanisms. In particular, essential oils and extracts obtained from plants such as thyme, hops, and rosemary significantly slow down the oxidative degradation of oils thanks to the protective effects of these components [67,69].

**Figure 2:**
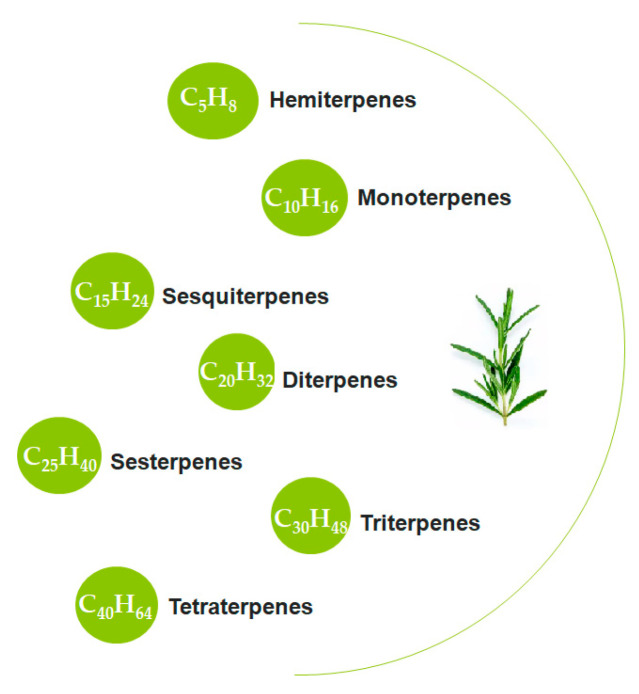
Classification of terpenes.

Terpenes are characterized as hydrocarbons with the general chemical formula (C_5_H_8_)_n_, which consists of repeating five-carbon (C_5_) isoprene units, and their oxygenated derivatives (Figure 2). One of the main mechanisms of action of terpenes is based on the “chain-breaking” process [69]. Terpene components such as carnosic acid and carnosol in rosemary extract act as free radical neutralizers in oil-based systems [67]. These natural compounds stop the chain reactions leading to oxidative degradation of oils and thus prevent the formation of harmful oxidation products [67]. In some studies, carnosic acid has shown a stronger protective effect compared to synthetic antioxidants such as BHA and BHT [68]. Another mechanism utilized by terpenes is the “singlet oxygen quenching” process. In this process, the terpene compound absorbs the excess energy of high-energy reactive oxygen and converts it into a lower-energy, stable form. Thus, the progression of oxidative degradation is prevented [65].

The antioxidant defense mechanism of terpenes occurs via the Nrf2–Keap1–ARE pathway. Under normal conditions, the Nrf2 protein is retained in the cytoplasm by Keap1 and degraded via the proteasome. Terpenes cause chemical changes in the critical cysteine regions of Keap1, preventing the degradation of Nrf2. As a result of this process, Nrf2 accumulates and is transported to the nucleus. Upon reaching the nucleus, Nrf2 binds to ARE sequences and increases the transcription of antioxidant enzymes such as HO-1, NQO1, SOD, catalase, and glutathione peroxidase. Thus, cellular redox balance is maintained, oxidative stress is reduced, and inflammatory processes are suppressed [70].

Among the mechanisms of action of terpenes, more specific processes are also involved. Some terpenes terminate the degradation reaction through a method called “termination enhancing” [71]. For example, the terpene γ-terpinene shows a highly unique mode of action known as the “slingshot” mechanism [72,73]. In this process, γ-terpinene is transformed into an unstable molecule by losing a hydrogen atom in the first step [72]. This unstable molecule then undergoes a structural rearrangement into a p-cymene form and releases a small, water-soluble radical called HOO- into the environment [72,73]. The HOO- radical moves rapidly from the depths of the oil phase to the external environment and stops the progression of the reaction by interfering with the oxidative degradation chain from the outside [72]. The most remarkable aspect of this “slingshot” mechanism is that, unlike vitamin E, γ-terpinene does not acquire pro-oxidant properties even when used at high concentrations [73].

These protection mechanisms have been found to be directly applicable in food production. For example, when rosemary extract (RLE) was added to products such as salmon paste, the formation of harmful MDA and cholesterol-derived degradation products was prevented, and the natural color of the product was preserved [67]. Similarly, the addition of thyme and hops oils to peanut oil leads to a decrease in peroxide values and increases the oxidative resistance of the oil [69].

Due to the lipophilic character of terpenes, they sometimes need to be stabilized in “carrier systems” in order to function more effectively in foods. In these carrier systems, thick protective layers of biopolymers are formed around the oil droplets [66,74]. These biopolymer layers physically limit the access of oxygen and other degradation-accelerating agents to the oil, thus enhancing the protective activity of terpenes and extending the shelf life of the product [74,75]. The role of terpenes as antioxidants is shown in Table 4.

## 6. Health Benefits of Plant-Derived Bioactive Compounds

The observed antioxidant and anti-inflammatory effects of plant-derived bioactive compounds in relation to meaningful health benefits as well as their mechanism of action are illustrated in Table 5.

## 7. Encapsulation and Delivery Systems of Plant-Derived Bioactive Compounds

Encapsulation and delivery systems of plant-derived bioactive compounds are important advancements in improving the antioxidant effectiveness by preventing degradation throughout processing, storage, and digestion [93]. Encapsulation of polyphenols in nanocarriers such as liposomes and nanoemulsions contributes not only to their stability and bioavailability, but also to their use in food applications as natural preservatives, satisfying consumers’ demand for safer and clean-label products. Meat and dairy products as well as beverages can constitute suitable food matrices for integrating BACs as antioxidants.

In brief, encapsulation refers to entrapping one material within another, and the resulting particles are characterized by sizes ranging from a few nanometers to a few millimeters [94]. Several approaches for the encapsulation of BACs have been developed to overcome some limitations regarding their bioavailability and food application. The choice of a suitable coating material is critical in encapsulation. The material must meet certain requirements, such as mechanical resistance, protection of the encapsulated material from degradation, and enabling the regulated release of the core substance. Recent studies on polyphenol encapsulation techniques, including their related advantages and disadvantages, are synthesized in Table 6.

Food-grade encapsulated polyphenols have gained great attention as novel food additives, both in food production and storage. They can act as self-life enhancer type additives. Some examples are follows: anthocyanins in desserts, milk and yogurt; flavonoids in sunflower oil and beef; resveratrol in fish oil; and catechin in bacon. Encapsulated polyphenols are applied as novel enhancers of physicochemical properties based on some major properties such as emulsifying (anthocyanin in pastry cream), water retention (anthocyanin in jelly candy), gelling capacity (flavonoids in meat batter and jelly), and thickening properties (polyphenols in ice cream and yogurt). A variety of sensory enhancers is used by food producers to enhancing consumers’ perception and acceptance of foods. Encapsulated polyphenols are gaining attention in recent years as types of additives that enhance sensory properties. In practical terms, they are used as colorants (anthocyanin in pastry cream, dairy products, cookie dough, and gummy candy), flavoring agents in baking food systems, and as colorant and flavoring agents (flavonoids in cookie production). In addition, encapsulated polyphenols can act as novel nutrition/function enhancers. For example, polyphenols have been recognized as nutrition fortifiers in dairy products, snacks, and fish burgers [110].

## 8. Applications in Food Engineering and Health

Flavonoids and terpenes are among the main phytochemical groups that enhance both technological performance and biofunctional properties in food engineering. Thanks to their antioxidant and antimicrobial properties, these compounds slow down lipid oxidation, contribute to the preservation of color and aroma, and extend the shelf life of products. These effects are particularly pronounced in meat, fish, and oil-based food matrices, where oxidative degradation is inhibited [2]. Polyphenols and flavonoids play an important role in reducing oxidative stress-induced cellular damage, regulating the immune response, and promoting human health, partly through antihypertensive mechanisms. Current research suggests that these compounds enhance product safety through their bioprotective properties and offer potential for functional food development strategies [111,112]. It has been determined that terpenes and polyphenols constitute an alternative to synthetic additives as natural preservative components and can be used to develop a preservation approach in line with consumer expectations [113].

**Figure 3:**
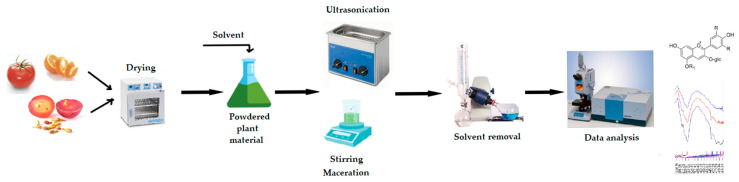
Extraction of plant-derived bioactive compounds. The direction of the arrows indicates the order of the various operations needed for the extraction and analysis of bioactive compounds. In brief, the plant material is subjected to drying under conditions that protect the compounds of interest, which are heat-sensitive (e.g., by lyophilization). Liquid solvents (water, alcohol, glycerin, etc.) are used and the extraction methods applied include classical techniques (maceration) or/and modern techniques (e.g., ultrasonication). The extracts are subjected to solvent removal stages, such as (micro)filtration, centrifugation or solvent vacuum evaporation. Various techniques ranging from spectroscopic methods to novel chromatographic techniques are employed further, for the identification and quantification of the bioactive compounds by interest.

A significant part of the technological value of these compounds comes from the rational choice of processing and extraction strategies (Figure 3). Modern technologies such as high hydrostatic pressure, pulsed electric fields, ultrasound, and ohmic heating increase cell wall permeability and facilitate the liberation of bound phenolic compounds. This process also contributes to the preservation of the total amount of phenolic compounds [114]. The adoption of optimization-oriented process designs with the use of green solvents helps to reduce the environmental burden and increases the yield [31,115]. Enzymatic hydrolysis and fermentation applications reduce bioaccessibility limitations and transform phenolic compounds from a bound form into more easily absorbable structures [116]. In addition, encapsulation methods and biopolymer-based carrier systems maintain the stability of compounds under gastrointestinal conditions and offer controlled release to the target site [116]. Engineering-based design of polyphenol–protein complexes increases the stability and target transportability of these compounds, thus strengthening the effectiveness of their functional effects [117].

Polyphenol–protein interactions at the formulation level influence not only bioavailability but also sensory and structural parameters. Polyphenols pre-aggregated with proteins prolong the half-life of foam drainage, increase structural strength, and contribute to a reduced perception of shrinkage. This also positively affects the flavor acceptance of the product, and this complexation mechanism may even lead to the masking of certain allergenic epitopes, leading to a decrease in allergenicity indicators [118]. However, in some matrices, polyphenols can have binding effects on protein digestibility and mineral absorption, so dosage and carrier system design should be carefully managed [119]. It has been reported that polyphenol–protein conjugates maintain their functionality through controlled release and resistance to oxidative degradation mechanisms, thus providing a balanced optimization between shelf life and biofunctional activity [117,120]. These approaches are considered as important formulation strategies that support the sustainability of the biological effect while maintaining the targeted sensory profile in food products [118].

**Figure 4:**
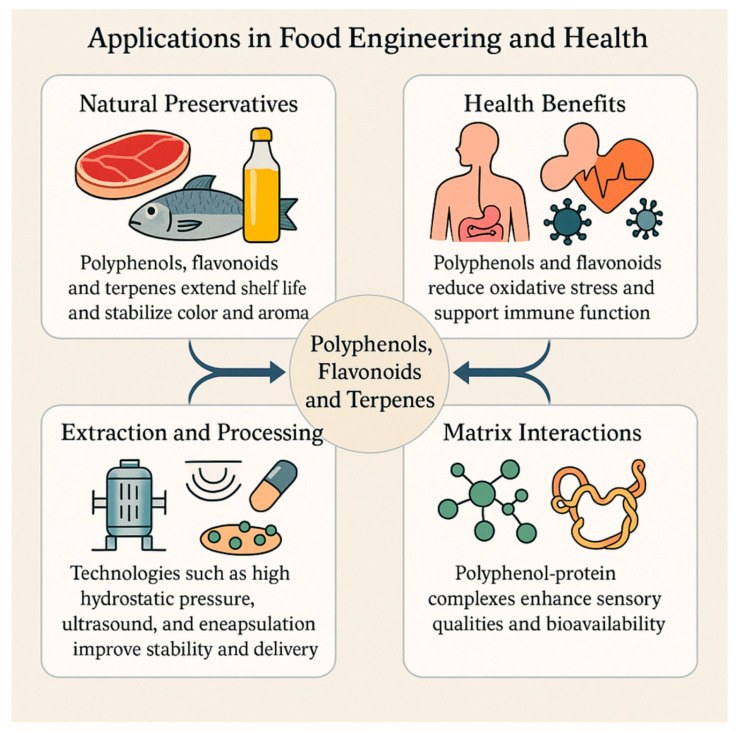
Applications of polyphenols, flavonoids and terpenes in food engineering and health.

In terms of health-oriented contributions, these compounds have a wide range of effects. Polyphenols and flavonols contribute to the strengthening of the antioxidant defense system and maintenance of mucosal immune balance by regulating the composition and metabolic activities of the gut microbiota [121]. Biofortification applications and genomics-based agricultural strategies enable the development of polyphenol-rich food raw materials and provide a scientific basis for nutritional solutions, especially for the reduction of intestinal inflammatory processes [122]. In vitro studies and early clinical data have shown that polyphenols have antimicrobial effects as well as inhibition potential on specific viral targets, providing dual benefits in terms of both food safety and consumer health [111]. The sustainable production of terpenes using yeast-based metabolic engineering approaches increases the supply of natural preservative resources and provides a higher level of flexibility to formulation processes [113]. This holistic picture (Figure 4) suggests that the combined evaluation of process and carrier system designs may contribute to the improvement of bioaccessibility, thereby reducing clinical effect differences and strengthening the scientific basis of functional foods [116,123].

In conclusion, polyphenols, flavonoids, and terpenes have important functions in delaying oxidative and microbial degradation in food systems, preserving tissue integrity and sensory properties, as well as favorable effects on gut microbiota, inflammation processes, and cardiometabolic indicators. Process innovations and green extraction approaches enable efficient transport and controlled release of these compounds by maintaining their stability throughout the product life cycle [31,114]. Encapsulation systems with protein-based complexes contribute to the management of matrix interactions and thus offer strategic advantages in terms of both technological efficiency and biofunctional performance [118,120]. Rational management of dosage, safety parameters, and possible anti-nutritional effects is critical for the sustainability of applications and the continuity of consumer benefit [117,119]. The scaling up of natural preservative production through biotechnological methods and the efficient utilization of agricultural by-products strengthen industrial viability and demonstrate a structural fit with circular economy principles [112,113].

## 9. Conclusions

Oxidative stress is a fundamental mechanism that threatens both human health and food quality as an imbalance between reactive oxygen species (ROS) and antioxidant capacity. In human physiology, it leads to the damage of cellular macromolecules (lipids, proteins, DNA) and paves the way for chronic conditions such as neurodegenerative and cardiovascular diseases. In the food industry, oxidative processes, especially through lipid oxidation, cause a reduction in the shelf life of products, unwanted taste and odor development, and the loss of nutritional value. Plant-derived bioactive compounds such as polyphenols, flavonoids, and terpenes play important roles in both food preservation and physiological health protection by providing powerful natural defense mechanisms against this bidirectional oxidative damage.

The activity of these bioactive compounds is based on multiple and interrelated biochemical pathways. Polyphenols and flavonoids have the capacity to directly scavenge free radicals and donate electrons due to their chemical structure (e.g., catechol groups). However, their activity is further enhanced by indirect mechanisms: They enhance the body’s own antioxidant enzymes (SOD, CAT, GPx4) by activating the Nrf2–Keap1 signaling pathway and suppress inflammatory responses through NF-kB inhibition. Terpenes, especially in lipophilic (oily) food matrices, effectively stop oxidative degradation through specialized methods such as chain-breaking reactions mediated by compounds such as carnosic acid and the “slingshot” mechanism of γ-terpinene. Specifically, while polyphenols and flavonoids primarily utilize enzymatic modulation and direct scavenging, terpenes employ distinct physical and chemical shielding strategies in lipid matrices, demonstrating how molecular mechanisms differ across bioactive classes.

From a food engineering perspective, these plant-derived bioactive compounds (BPCs) represent a powerful alternative to synthetic antioxidants (BHA and BHT), meeting consumer demands for “clean-labeled” products. In terms of effectiveness and safety, natural compounds such as carnosic acid compare favorably to their synthetic counterparts, offering potent oxidative protection without the potential health risks associated with synthetic antioxidants. They retard lipid peroxidation and the formation of secondary spoilage products such as malondialdehyde (MDA) in meat, fish, and oil-based products, preserving the flavor, color, and nutrient profile of foods. The activity of these compounds in food matrices is enhanced by modern processing and formulation techniques such as encapsulation, biopolymer carriers, and the formation of polyphenol–protein complexes. These approaches optimize the stability, bioaccessibility, and sensory acceptance of BACs. However, while encapsulation and carrier systems enhance functional stability and sensory properties, they may present disadvantages such as interactions with protein digestibility, necessitating careful dosage management.

In conclusion, flavonoids and terpenes are versatile compounds that offer protective effects on human health while enhancing stability in food systems. Beyond their technological applications, these compounds also have the potential to modulate gut microbiota and support immune balance. To translate preclinical findings into meaningful health benefits for human populations, particularly in neurodegenerative diseases, strategies to overcome variations in bioaccessibility and enhance absorption in the gastrointestinal tract must be prioritized. Future research should focus on the optimization of these delivery systems to improve bioaccessibility and thus clinical efficacy. Furthermore, the development of green extraction methods such as high hydrostatic pressure or ultrasound and the utilization of agricultural by-products (e.g., food processing wastes) as a source for these valuable compounds will secure the sustainable use of BACs in the food and health fields. These sustainable production and extraction methods align with circular economy principles by valorizing waste streams, thereby significantly reducing the environmental impact of food production.

## Figures and Tables

**Table 1:** Antioxidant protection mechanisms existing in the human body.

Category	Representatives	Specific Action
Preventative antioxidant enzymes	Superoxide dismutase (SOD)	Converts the superoxide anion radical into hydrogen peroxide
	Catalase	Scavenges hydrogen peroxide, yielding water and molecular oxygen by decomposing H_2_O_2_
	Glutathione peroxidase	Decomposes hydrogen peroxide and hydroperoxides at the expense of glutathione
Primary antioxidants (chain-breaking antioxidants)	α-Tocopherol Catechins Phenolic antioxidants	Retards initiation or interrupts propagation Converts radical species into more stable radicals or non-radical species
Secondary antioxidants	Metal chelators Oxidative enzyme inhibitors	Decomposes peroxides, yielding non-radical species Chelates prooxidative metal ions Inhibits oxidative enzymes Absorbs UV radiation
Complementary agents	Ascorbic acid β-carotene Retinoids	Scavenges active oxygen and free radicals

**Table 2:** Different mechanisms of action of polyphenols in relation to their antioxidant properties.

Mechanism	Main Effect	Key References
Direct scavenging of ROS/RNS	Neutralizes hydroxyl radicals and superoxide anions to prevent cellular damage	[33,34]
Metal ion chelation	Reduces free radical formation by binding to metal ions	[34]
Suppression of oxidative enzymes	Decreases ROS production by inhibiting oxidative enzymes such as xanthine oxidoreductase	[36]
Activation of the Nrf2–Keap1 pathway	Increases synthesis of antioxidant enzymes like SOD, catalase, and GPx4	[35,40]
Inhibition of NF-κB pathway	Reduces inflammatory cytokine synthesis by blocking gene transcription	[34,36]
Regulation of PI3K–Akt and IL-17 signaling	Regulates stress response proteins, leading to anti-inflammatory effects	[42,43]
Maintenance of mitochondrial function	Protects mitochondrial DNA and enhances energy production	[37,44]
Induction of autophagy	Clears damaged organelles and promotes cellular renewal	[4]
Regulation of nitric oxide homeostasis	Balances eNOS activity and enhances vascular function	[36,43]

**Table 3:** Different mechanisms of action of flavonoids in relation to their antioxidant properties.

Mechanism	Main Effect	Key References
Direct scavenging of reactive oxygen species (ROS)	Interrupts free radical chain reactions by donating electrons or hydrogen atoms	[55,56,57]
Structural determinants of antioxidant activity	Hydroxyl and catechol groups enable strong binding to hydroxyl radicals; gallate groups enhance activity	[58,59]
Regulation of enzymatic antioxidant systems	Increases the activity of SOD, CAT, and GPx enzymes to detoxify reactive oxygen species	[56,60,61]
Activation of the Nrf2 signaling pathway	Stimulates the transcription factor Nrf2, leading to upregulation of antioxidant genes and enzyme synthesis	[60,62]
Restoration of cellular redox balance and mitochondrial protection	Reduces intracellular ROS and MDA levels, restores mitochondrial membrane integrity, and enhances cell resistance	[55,63,64]

**Table 4:** Different mechanisms of action of terpenes in relation to their antioxidant properties.

Mechanism	Main Effect	Key References
Chain-breaking antioxidant action	Stops lipid oxidation chain reactions by neutralizing free radicals in oils (e.g., carnosic acid, carnosol)	[67,69]
Singlet oxygen quenching	Converts high-energy singlet oxygen into stable molecular oxygen, preventing oxidative degradation	[65]
Termination enhancing mechanism	Accelerates the end of oxidation chain reactions by promoting stable product formation	[71]
Slingshot mechanism (γ-terpinene)	Releases water-soluble HOO• radicals that halt oxidation externally without pro-oxidant effect	[72,73]
Application in food preservation	Prevents MDA and cholesterol oxidation products formation, maintaining food color and quality	[67,69]
Encapsulation in biopolymer carrier systems	Enhances terpene stability by forming biopolymer barriers that limit oxygen access and prolong shelf life	[66,74,75]

**Table 5:** Selected health benefits of plant-derived bioactive compounds.

BACs	Health Benefits	Mechanism of Action	References
Cardamonin	Antiangiogenic and anticancer effects	Suppressing mammalian target of rapamycin as well as vascular endothelial growth factor (VEGF)-induced angiogenesis via microRNAs	[76]
Flavonoids from grape seed extract	Protects human blood lymphocytes against oxidative stress induced by ionizing radiation	Significant decrease in the incidence of micronuclei protection	[77]
Flavonoid and total polyphenols	Anticarcinogenic potential	Inhibition of the production of NO and TNF-α	[78]
Gallic acid	Induces cancer cell death	Inhibition of histone deacetylase	[79]
Caffeic acid	Cell cycle arrest in oral cavity, neck, and tongue carcinoma cell lines	Modulates key signaling pathways such as NF-kβ, MAPK, and AKT (protein kinase B)	[80,81]
Phenolic-rich water extract of napiergrass	Protection to BNL cells from H_2_O_2_-induced cytotoxicity	Upregulates the levels of GSH and that of antioxidant enzymes	[82]
Lettuce polyphenols	Antidiabetic effects	Inhibition of hepatic glucose-6-phosphate translocase, lower fasting blood glucose in *db/db* mice	[83]
Grape pomace extract (phenolic compounds)	Gut health	Selective modulation of the rat gut microbiota to a healthier phenotype	[84]
Cranberry polyphenols	Gut health	Improves high fat/high sucrose diet-induced features of the metabolic syndrome, with a proportional increase in the *Akkermansia* spp. population	[85]
Adzuki bean polyphenols	Gut health	Reduces butyrate production	[86]
Flavonoids from *M. paradisiaca*	Reduction of cholesterol levels	Reduces the activities of lipogenic enzymes Increases turnover of cholesterol into bile acids and neutral sterols	[87]
*Idesia polycarpa* polyphenols	Lipid-lowering effect	Activates PPARα in association with decreased expression of NF-κB, and IL-1	[88]
Grape polyphenols	Improves vascular function	Potentiates vasorelaxation and reduces BP and circulating cell adhesion molecules	[89]
Brown algae (*Ecklonia cava*) polyphenols	Prevents tumor progression in vivo	Inhibits the activity of cyclooxygenase-2 and cell proliferation	[90]
Lemon seeds flavonoids	Improvement of oxidative stress system	Activation of the Nrf2 antioxidant signal pathways	[91]
Resveratrol from red wine	Improving brain health	Inhibition of beta amyloid protein aggregation	[92]
Diterpene from red alga *Gracilaria edulis*	Antiproliferative activity against the human lung adenocarcinoma cell line A549	Several mechanisms of action, such as apoptosis	[90]

**Table 6:** Selected studies on the encapsulation of some plant-derived bioactive compounds.

BACs	Wall Material	Advantages/Disadvantages	Impact in Food Matrix/Food Application	Ref.
Spray drying technique				
Gallic acid	Bacterial exopolysaccharide	No antioxidant activity was lost during the encapsulation process/different release profiles of the bioactive compounds in simulated gastric and intestinal fluids	To be investigated	[95]
Chlorogenic acid	Maltodextrin	Powders with good chemical and physical properties/-	To be investigated	[96]
Anthocyanins	Maltodextrin	The shelf life and stability of spray-dried blackberry anthocyanins was greatly enhanced by the presence of copigments/poor stability and rapid loss of coloring properties	Formation of colored derivatives and polymers during storage due to the degradation of anthocyanins	[97]
Anthocyanins	Gum arabic, n-octenyl succinic anhydride-modified starch	Highest encapsulation efficiency/-	No data	[98]
Anthocyanins	n-Octenyl succinic anhydride modified starch, low-viscosity gum arabic alternative	Greatest storage stability/-	No data	[98]
Grape skin phenolics	Maltodextrin	Good processing and storage stability/-	Potential sustainable, beneficial coloring agents and health promoting compounds	[99]
Blueberry polyphenols	Whole wheat flour, soy protein isolate, chickpea flour, coconut flour	Very positive performance on all in vitro biological assays/-	Novel environmentally-friendly anthocyanin-rich dietary sources with promising application as healthy ingredient	[100]
Freeze-drying technique				
Phenolic compounds from spent coffee grounds	Maltodextrin, gum arabic	Maltodextrin as good option for encapsulation of antioxidant phenolic compounds/detrimental effect of gum arabic on the retention of phenolics, as well as on the antioxidant activity	No data	[101]
Polyphenols from red wine	Maltodextrin, gum arabic	Phenolic concentration of the dealcoholized wine powder is 7.1 times higher than the original liquid red wine; good stability of wine powder over 145 days under accelerated storage conditions; protection to phenolics against oxidation/an important decrease in malvidin 3-G compared to caffeic acid and resveratrol	Polyphenol enrichment of healthy powder drinks with wine powder	[102]
Polyphenols from *Elsholtzia ciliate* herb extract	Skim milk, maltodextrin, resistant-maltodextrin, sodium caseinate, gum arabic, beta-cyclodextrin	High encapsulation efficiency of polyphenols in sodium caseinate and in mixture with resistant-maltodextrin and maltodextrin, respectively/lower encapsulation efficiency of polyphenols with maltodextrin	Freeze-dried powders could be incorporated in food	[103]
Phenolic and volatile compounds of raspberry juice	Brown rice proteins	Highest phenolic content, anthocyanin content, and antioxidant activity in complexes with the lowest amount of protein (2%)/changes in the denaturation temperature of complexes	Novel food ingredient with health benefits and sensory attributes, potential to be used as food colorant and flavoring	[104]
Extrusion				
Extract of *Hibiscus sabdariffa* L.	Double emulsion (hibiscus extract/rapeseed oil/pectin) and a cross-linked solution (CaCl_2_)	Microparticles with greater stability of anthocyanins and color; higher bioactive compound retention in yogurt matrix/less homogeneous color distribution	Microparticles incorporated in the yogurt matrix promote lactic acid bacteria viability, color, and functionality	[105]
Emulsification				
Quercetin in combination with other antioxidants from natural sources	Oil-in-water (O/W) nanoemulsions	Inhibition of polyphenol oxidase/turbidity of nanoemulsions, without affecting their stability	Controlling of enzymatic browning of apples	[106]
Microfluidic technology				
Curcumin and catechin	Dipalmitoylphosphatidylcholine	Encapsulation efficiencies of curcumin and catechin in the dual-loaded liposomes were 100% and 16.77%, respectively/-	Liposomal encapsulation of functional compounds	[107]
Supercritical antisolvent technology				
*Trans*-resveratrol	Ethanol/dichloromethane mixture	Enhanced oral bioavailability of *trans*-resveratrol; nanoparticles with particle size controlled by solvent composition/-	Pure *trans*-resveratrol nanoparticles for the development of health supplements	[108]
Electrostatic-encapsulation technologies/electrospraying				
Polyphenols from maqui fruit extract	Cyclodextrin	Improved thermal stability of polyphenols; preserved total phenolic content at high-temperature treatments/-	Food formulations involving high temperatures, such as bakery and dairy products	[109]

## Data Availability

No new data were created or analyzed in this study. Data sharing is not applicable to this article.
